# Chlorine disinfection promotes the exchange of antibiotic resistance genes across bacterial genera by natural transformation

**DOI:** 10.1038/s41396-020-0656-9

**Published:** 2020-04-23

**Authors:** Min Jin, Lu Liu, Da-ning Wang, Dong Yang, Wei-li Liu, Jing Yin, Zhong-wei Yang, Hua-ran Wang, Zhi-gang Qiu, Zhi-qiang Shen, Dan-yang Shi, Hai-bei Li, Jian-hua Guo, Jun-wen Li

**Affiliations:** 1 Department of Environment and Health, Tianjin Institute of Environmental & Operational Medicine, Key Laboratory of Risk Assessment and Control for Environment & Food Safety, No 1 Dali Road, Tianjin, 300050 PR China; 2 Advanced Water Management Centre (AWMC), University of Queensland, St Lucia, Brisbane, QLD 4072 Australia

**Keywords:** Public health, Water microbiology

## Abstract

Chlorine disinfection to drinking water plays an important role in preventing and controlling waterborne disease outbreaks globally. Nevertheless, little is known about why it enriches the antibiotic resistance genes (ARGs) in bacteria after chlorination. Here, ARGs released from killed antibiotic-resistant bacteria (ARB), and culturable chlorine-injured bacteria produced in the chlorination process as the recipient, were investigated to determine their contribution to the horizontal transfer of ARGs during disinfection treatment. We discovered *Esch*e*richia coli*, *Salmonella aberdeen*, *Pseudomonas aeruginosa* and *Enterococcus faecalis* showed diverse resistance to sodium hypochlorite, and transferable RP4 could be released from killed sensitive donor consistently. Meanwhile, the survival of chlorine-tolerant injured bacteria with enhanced cell membrane permeabilisation and a strong oxidative stress-response demonstrated that a physiologically competent cell could be transferred by RP4 with an improved transformation frequency of up to 550 times compared with the corresponding untreated bacteria. Furthermore, the water quality factors involving chemical oxygen demand (COD_Mn_), ammonium nitrogen and metal ions (Ca^2+^ and K^+^) could significantly promote above transformation frequency of released RP4 into injured *E. faecalis*. Our findings demonstrated that the chlorination process promoted the horizontal transfer of plasmids by natural transformation, which resulted in the exchange of ARGs across bacterial genera and the emergence of new ARB, as well as the transfer of chlorine-injured opportunistic pathogen from non-ARB to ARB. Considering that the transfer elements were quite resistant to degradation through disinfection, this situation poses a potential risk to public health.

## Introduction

Antibiotic resistance has become a worldwide crisis. It is estimated that antibiotic resistance will cause as many as 10 million casualties annually by 2050 if no action is taken now [1–4]. Meanwhile, as increasing kinds of antibiotic resistance genes (ARGs) and antibiotic-resistant bacteria (ARB) are discovered in aquatic ecosystems [5–7], environmental ARGs that may promote the global transmission of ARB have been classified as emerging pollutants. Therefore, approaches to help disseminate ARGs in the environment are attracting global attention [8–15].

As a widely used tool to kill pathogens and ensure the microbiological safety of drinking water, chlorine disinfection has made a great contribution to preventing and controlling waterborne disease outbreaks [16–18]. Theoretically, it should help diminish, or even eliminate, ARB and ARG. However, the reported enrichment of the ARG level in the finished water after chlorination means a much higher proportion of either intracellular ARG (iARG) or extracellular ARG (eARG) in post-disinfection water than that in pre-disinfection water [19–22]. This realisation that chlorine disinfection enriches the ARG in bacteria and promotes the transmission of ARB and ARG in water poses a potential risk to public health. Interestingly, the reason for this and the factors that trigger antibiotic resistance dissemination during disinfection remain unclear.

Natural genetic transformation is a process in which a competent bacterial recipient takes up naked DNA and incorporates it into its own chromosome or converts it into an autonomous extra-chromosomal replicon [23]. As an important approach for spreading ARG, besides the competent bacterial recipient, a donor is required to provide the ARG for transfer. Considering that a large number of ARG carriers with biological activity would be released from the killed donors during the chlorination process [24], which would contribute to increasing eARG after disinfection [25], it has been hypothesised that the chlorination process would enhance iARG abundance by promoting the uptake of released ARG and genetic transformation across bacteria. Furthermore, culturable chlorine-injured bacteria may play roles in this process. Culturable chlorine-injured bacteria are viable but physiologically unhealthy populations tolerant to chlorine. These bacteria may account for as much as 90% of all indicator bacteria present after disinfection [26]. Due to suffering from reversible damage as a consequence of partial or inappropriate disinfection, they are undetectable using specific selective media, resulting in the underestimation of their presence, and health can be recovered only in certain circumstances, thereby posing hazards for water safety [13, 27]. Previously, we found temporal physiological persistence to antibiotics in culturable chlorine-injured *Pseudomonas aeruginosa* [28]. However, the contribution of cultivated chlorine-injured bacteria to genetic transformation during the treatment of disinfection remained unclear.

Herein, after the exposure of sodium hypochlorite (NaClO) to *Escherichia coli*, *Salmonella aberdeen*, *P. aeruginosa* and *Enterococcus faecalis* with plasmid RP4, the transferability of RP4 released from killed ARB (donor) and their genetic transformability to chlorine-injured bacteria (recipient) were observed. Then, the effects of physicochemical parameters, including pH, temperature, chemical oxygen demand (COD_Mn_), ammonium nitrogen (NH_4_^+^–N) and metal ions (Ca^2+^ and K^+^) on the natural transformation frequency of released plasmid RP4 from killed bacteria into chlorine-injured *E. faecalis*, which are frequent causes of biofilm-associated opportunistic infections [29, 30], were further explored. For the first time, we showed that cultivated chlorine-tolerant injured non-ARB, which was a kind of competent cell with cell membrane permeabilisation and a strong oxidative stress-response, could uptake released RP4 from sensitive donors and then shift into ARB persistently with a higher frequency. This information will help in comprehending the reasons for the enrichment of the ARG level during the chlorination process and reveal the dissemination approaches of ARGs between bacteria during the disinfection process.

## Materials and methods

### Bacterial strains and media

A list of the bacterial strains and plasmids used in this study is given in Supplementary Table S1. The culture conditions of the bacteria are shown in Supplementary Text 1.

### Disinfection experiments

Microbial inactivation experiments were conducted in sterile 250-mL glass bottles containing 100 mL of bacterial suspension at a final concentration of 10^5^–10^6^ cfu/mL. The preparation of the bacterial suspension for the exposure of NaClO (Sigma–Aldrich, USA) is shown in Supplementary Text 2. NaClO was then added to all vials (except for the control vials), and the samples were mixed well in a shaker at 150 rpm. Ten mililitres samples was collected before and after NaClO contact with the bacteria suspension at various time points (i.e., 15 s, and 1, 2, 5, 10, 20 and 30 min). All the samples treated with NaClO were immediately neutralised with 0.1 mol/L of sodium thiosulphate and then analysed for residual chlorine using the *N*,*N*-diethyl-*p*-phenylenediamine method [31]. Each experiment was performed, in triplicate, in a temperature-controlled incubator. The details of the disinfection kinetic modelling of NaClO are given in Supplementary Text 3.

### Numeration of injured bacteria

The number of viable injured bacteria in the treated water samples was determined, according to the previous method [5, 6, 13, 28]. Briefly, appropriate dilutions of bacterial samples treated with NaClO were spread on TSYA and corresponding selection plates, in triplicate (Supplementary Text 1). After overnight incubation at 37 °C, both the number of viable bacteria on the TSYA and uninjured bacteria on the selective medium were calculated. The difference value was determined to be the viable injured bacteria.

### Detection of transferable RP4 by genetic transformation

Functional transferable RP4 was detected as positive if the samples could transform chemically competent *E. coli* DH5α successfully through the heat-shock method [32]. DNA released in the disinfected samples at different time points was concentrated, according to Supplementary Text 4. Then, 10 μL of concentrated DNA samples was added to 50 μL of chemically competent *E. coli* DH5α (Takara, Dalian, China), which was placed on ice for 30 min before a heat-shock at 42 °C for 90 s. A total of 500 μL of SOC medium was added to each tube and incubated in a 37 °C water bath for 1 h. The number of transformants was determined by spreading aliquots of cell suspension on SOB plates containing 50 mg/L kanamycin (Kan), 60 mg/L ampicillin (Amp) and 40 mg/L tetracycline (Tet). Each experiment was performed in triplicate.

### Cell permeability assessment

The hydrolysis rate of the *o*-nitrophenyl-β-d-galactopyranoside (ONPG) by *E. coli* was measured to assess cell permeability [13] and the absorbance values at 420 nm were monitored. Each experiment was performed in triplicate. The details are shown in Supplementary Text 5 and  6.

### Bacterial reactive oxygen species (ROS) and antioxidant systems measurement

Bacterial ROS were detected with a DCF-DA/H_2_DCFDA-cellular ROS detection assay kit (Abcam, UK) and flow cytometry (BD FACS Calibur, USA). Bacterial antioxidant systems, including catalase (CAT) activity, superoxide dismutase (SOD) activity and glutathione peroxidase (GSH-Px) activity, were assayed with commercial kits from the Nanjing Jiancheng Bioengineering Institute (Nanjing, China) and a microplate reader (Molecular Devices, USA). All assays were done in compliance with the manufacturer’s instructions and were performed in triplicate. The details of these procedures are shown in Supplementary Text 5, 7 and  8.

### Assay of natural transformation induced by NaClO exposure

Considering the occurrence of conjugal transfer between the bacteria, two kinds of bacteria with different chlorine resistances were exposed to NaClO under the same conditions, separately, and then mixed in order to demonstrate the plasmid transformation induced by NaClO exposure. Briefly, water samples with 10^9^ cfu/mL of *E. coli*, *S. aberdeen* and *P. aeruginosa* (carrier of RP4, donors) were treated with 6 mg/L NaClO for 20 min to kill most cells (Supplementary Table S2) and then neutralised by 0.1 mol/L of sodium thiosulfate and filtered (0.22-μm filter, Millipore, USA) to remove the entire residual bacteria. In parallel, 6.3 × 10^8^ cfu/mL of chlorine-injured *E. faecalis* without RP4 (recipients) was prepared with 6 mg/L NaClO for 20 min (Supplementary Text 5). Then, 10 μL of the filtered solution was added to 100 μL of prepared injured bacteria and co-cultured at 37 °C for 60 min. Following this, the mixtures were spread on SOB medium containing 50 mg/L Kan, 60 mg/L Amp and 40 mg/L Tet. After overnight incubation at 37 °C, the colonies were counted. Each experiment was performed in triplicate.

In addition, to ensure that the bacteria growing on the SOB medium were transformers rather than any spontaneous mutations of the recipients or the contamination of donors, at least five colonies were spread on CATC plates of *E. faecalis* containing 50 mg/L Kan, 60 mg/L Amp and 40 mg/L Tet, and their plasmids were extracted to detect the specific *TraG* of RP4 by PCR. Detailed information about the RP4 detection by PCR is presented in Supplementary Table S3. The transformation frequency was calculated by dividing the number of transformants by the viable counts.

### Statistical analysis

Statistical analyses were performed using IBM SPSS Statistics (version 20.0, Armonk, NY: IBM Corp). The transformation frequency and oxidative stress-response of various groups were analysed using the Student’s *t* test. The difference of the ONPG hydrolysis curves between *E. coli* suffering from chlorine injury or not was determined by one-way ANOVA test. For all tests, only data resulting in *P* values < 0.05 were regarded as statistically significant.

## Results

### ARB presented diverse resistance to chlorine disinfection during NaClO exposure

To observe the dynamics of ARB killing, the inactivation kinetics of four kinds of ARB—*E. coli*, *P. aeruginosa*, *S. aberdeen* and *E. faecalis* (with RP4)—were investigated during bench-scale inactivation experiments with NaClO, which were carried out in buffered disinfectant demand-free water at pH 7.2 and 20 °C. Fitted chlorine decay curves and bacterial inactivation kinetics are shown in Supplementary Figs. S1–S3. Chlorine decay constants *k*′ with different initial NaClO concentrations, and the estimated efficiency factor Hom (EFH) model parameters are summarised in Supplementary Table S4. These data showed that the EFH model was a good fit for the inactivation data of all observed ARB, with *R*^*2*^ above 0.97. The *Ct* values (Supplementary Table S5), that is, the product of the disinfectant concentration and contact time needed to achieve a defined reduction of target organisms, were calculated by fitting the EFH model for different levels of ARB inactivation by NaClO.

**Fig. 1:**
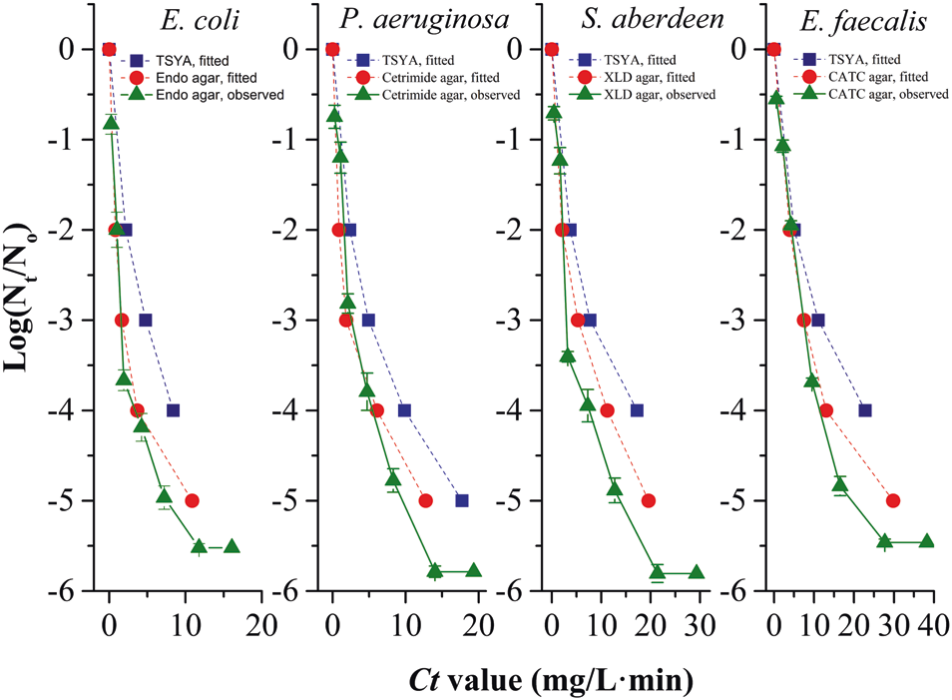
Inactivation curves of various antibiotic-resistant bacteria (ARB) during exposure to sodium hypochlorite (NaClO) when detected with selective medium and TSYA medium. The baseline conditions were as follows: the initial concentration of bacteria was 10^5^–10^6^ cfu/mL (pH 7.2, 20 °C). *N*_t_/*N*_o_: number of viable bacteria detected by TSYA medium or selective medium at a given time/number of bacteria at zero time. Dash lines, fitted *Ct* values by the estimated efficiency factor Hom (EFH) model. Solid line, observed *Ct* values (*n* = 3; mean ± SD).

The inactivation curves of observed ARB (Fig. 1) were based on the *Ct* value calculated by fitting the EFH model. A similar lethal bacterial tendency can be found whether it was detected by TSYA or a selective medium, but the lethal rate detected with a selective medium was higher than that with TSYA due to its detection failure of chlorine-injured bacteria. For example, after the exposure of 8.4 mg/L min, 4.0-log killing of *E. coli* could be observed for the TSYA medium, while it was above 4.5-log killing for the Endo media. Importantly, the strains exhibited diverse resistant abilities to NaClO, although *E. faecalis* was the strongest. To kill 4-log *E. faecalis* fully, the *Ct* values reached 22.8 mg/L min, which was detected with TSYA medium. Otherwise, *E. coli* was demonstrated to be the most sensitive against NaClO, among the observed bacteria. The *Ct* value to kill 4-log *E. coli* fully was only 8.4 mg/L min. Significant differences between the *Ct* values to kill various bacteria fully demonstrated that it is possible that bacteria with strong resistance to chlorine, such as *E. faecalis*, can survive NaClO exposure even if sensitive *E. coli* are fully killed.

### Killed ARB after NaClO exposure can donate transferable plasmids to surroundings

During the process of NaClO suffering, an increasing amount of killed ARB released the corresponding RP4 into the surroundings gradually. To test the transferability of RP4 released from ARB, ARB suspensions suffering from different doses of NaClO were filtered to remove ARB and then co-cultured with chemically competent *E. coli* DH5α. Table 1 summarises the *Ct* values for destroying the RP4 released from ARB at pH 7.2 and 20 °C. It showed that transformants could still be formed for the filtered water after disinfection even at *Ct* values of 25.8 mg/L min for *P. aeruginosa* and 39.8 mg/L min for *E. faecalis* when no viable ARB was detectable by the Cetrimide agar or CATC agar (Fig. 1). Therefore, transferable plasmids could be released from killed sensitive ARB, and much more NaClO was required to remove plasmids than was required to kill ARB (compare Table 1 and Fig. 1). Even if the ARB were killed, RP4, as an ARG carrier donated by the ARB present in water, was stable and remained transferable.

**Table 1:** Transformability of released RP4 from NaClO-treated ARB (10^5^ − 10^6^cfu/mL) to chemically competent *Escherichia coli* DH5α at pH 7.2, 20 °C.

ARB (with RP4)	Dose (mg/L)	Observed *Ct* value (mg/L min)							
		10.59	13.17	23.73	25.83	31.17	39.84	44.65	74.26
*E. coli*	6.0	+	/^a^	−	−	−	−	−	−
*P. aeruginosa*	6.5	+	+	+	+	/^a^	/^a^	−	−
*S. aberdeen*	7.5	+	+	/^a^	/^a^	−	−	−	−
*E. faecalis*	9.0	+	+	+	+	+	+	/^a^	−

+ transformant formed; − no transformant.

*P. aeruginosa Pseudomonas aeruginosa*; *S. aberdeen* *Salmonella aberdeen*; *E. faecalis* *Enterococcus faecalis*

^a^Not detected.

### Survival of chlorine-injured bacteria produced throughout NaClO exposure showed physiological competence with increased membrane permeability and a strong oxidative stress-response

**Fig. 2:**
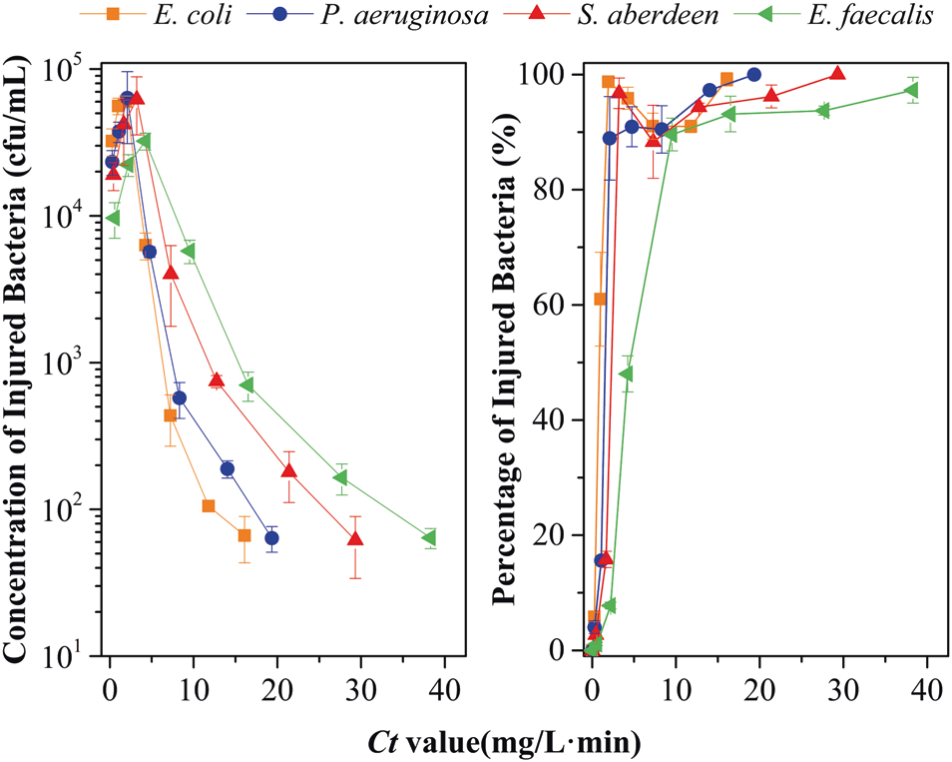
Production curves of chlorine-injured bacteria and their percentages of total viable bacteria during exposure to NaClO (*n* **=** 3; mean **±** SD). The baseline conditions were as follows: the initial concentration of bacteria was 10^5^–10^6^ cfu/mL (pH 7.2, 20 °C).

As a parallel, the production of chlorine-injured bacteria during the process of NaClO exposure was found continuously. From Fig. 2, the concentration of injured bacteria would peak at the 2-min exposure-time point, whose *Ct* values are in the range 2.0–4.2 mg/L min. Then, their concentration would decline gradually as the exposure dose increased. Otherwise, the percentage of injured bacteria in viable cells would increase sharply and all residual viable *E. coli*, *P. aeruginosa*, *S. aberdeen*, and 97% of viable *E. faecalis*, would become injured (below 100 cfu/mL) when exposure to NaClO reached 16.1, 19.3, 29.3 and 38.3 mg/L min, respectively. Therefore, during the chlorination process, viable bacteria became injured gradually and, ultimately, surviving populations with stronger tolerance to chlorine were injured.

**Fig. 3:**
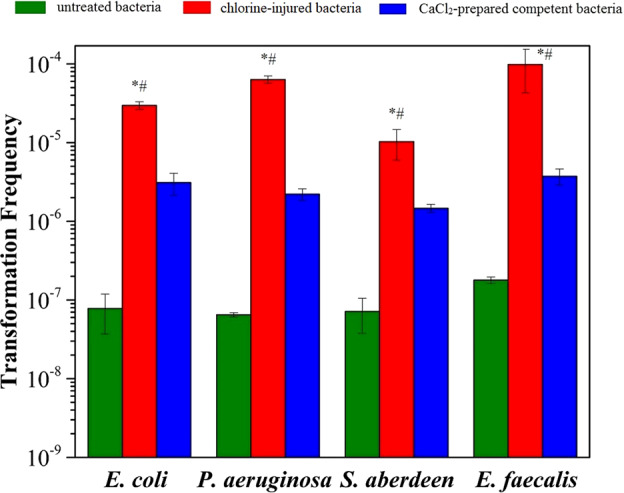
Comparison of RP4 transformability with chlorine-injured bacteria and CaCl_2_-prepared competent bacteria (*n* **=** 3; mean **±** SD). RP4 (100 ng) was added to 100 µL of 10^8^ cfu/mL chlorine-injured or competent bacteria prepared by the CaCl_2_ method, according to the heat-shock assay [32, 45]. Bacteria (10^8^ cfu/mL) without exposure to NaClO were selected as controls. *p < 0.01, compared with untreated bacteria; ^#^*p* < 0.01, compared with CaCl_2_-prepared competent bacteria.

To test if chlorine-injured bacteria were physiologically competent, RP4 was used to evaluate their transformability to chlorine-injured or CaCl_2_-prepared competent *E. coli*, *P. aeruginosa*, *S. aberdeen* and *E. faecalis* (Fig. 3, Supplementary Text 5). The data showed that RP4 could enter injured bacteria of all kinds of those mentioned above and survive in them successfully, presenting a more efficient transformation than corresponding CaCl_2_-prepared competent bacteria (*p* = 1.87 × 10^−4^, 1.01 × 10^−4^, 6.61 × 10^−3^, 1.88 × 10^−3^, respectively) and untreated ones (*p* = 1.0 × 10^−4^, 8.8 × 10^−5^, 3.3 × 10^−3^, 1.5 × 10^−3^, respectively). Above all, compared with the corresponding untreated bacteria, chlorine-injured *E. faecalis* improved the transformation frequency of RP4 by 550-fold, reaching 9.8 × 10^−5^, while it was only enhanced 21-fold for CaCl_2_-prepared competent bacteria, equating to a transformation frequency of 3.7 × 10^−6^.

**Fig. 4:**
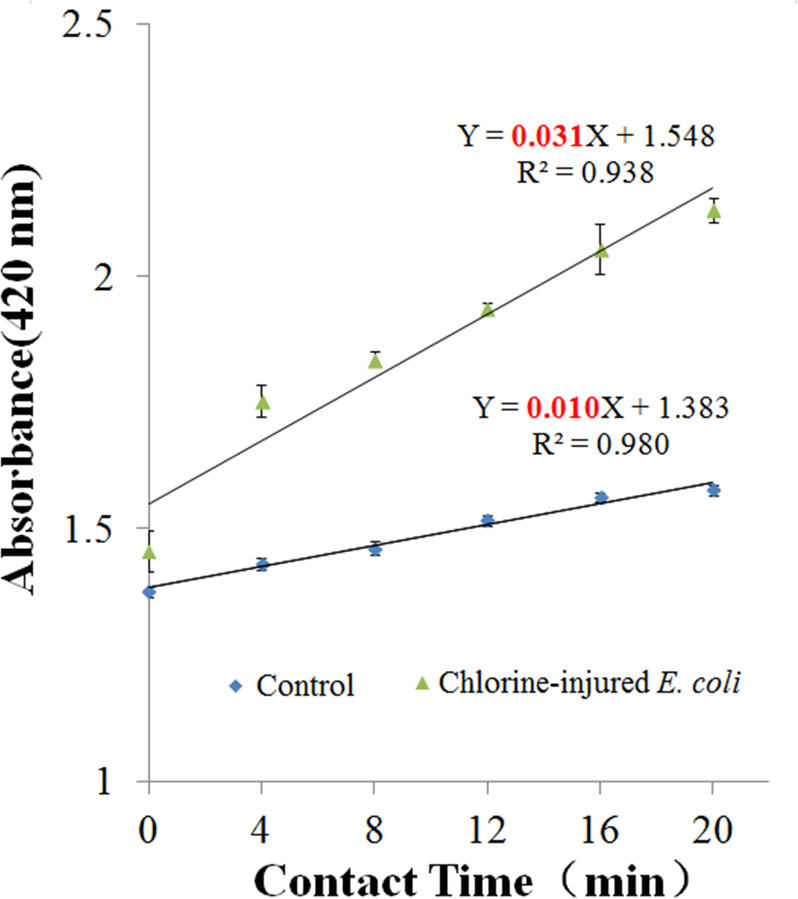
Observed and fitted ONPG hydrolysis curves for *Escherichia coli* suffering from chlorine injury or not (*n* **=** 3; mean **±** SD). The slope of the line (i.e., the rate of ONPG hydrolysis) was 0.010 and 0.031 for the control (*E. coli* without exposure) and chlorine-injured *E. coli*, respectively.

An ONPG hydrolysis rate of 10^8^ cfu/mL above chlorine-injured *E. coli* was exhibited (Fig. 4). Compared with the control (untreated *E. coli*), injured *E. coli* increased their hydrolysis rate of ONPG by a significant 3.1-fold (*p* = 0.0095). It indicated that the cell membrane had increased permeability in the chlorine-injured bacteria after the exposure to NaClO.

**Fig. 5:**
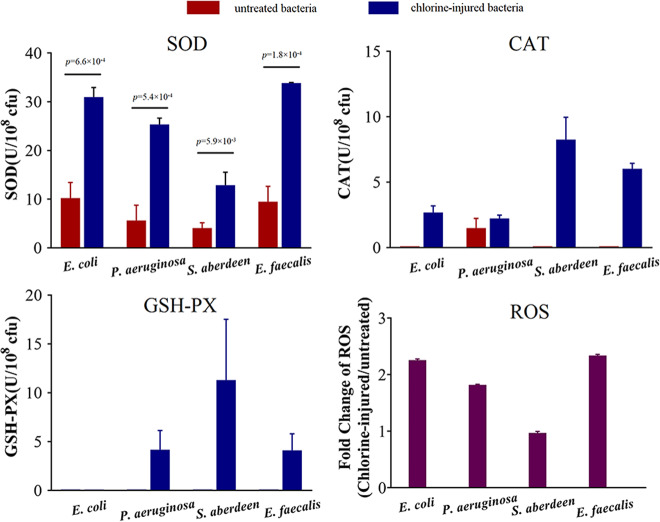
Oxidative stress-response in chlorine-injured bacteria or not at pH 7.2, 20 °C (*n* **=** 3; mean **±** SD). Bacterial suspensions (10^8^ cfu/mL), chlorine-injured or not, were sonicated at 20 kHz for 10 min and then centrifuged at 5000 × *g*, 4 °C for 3 min. The levels of superoxide dismutase (SOD), catalase (CAT) and glutathione peroxidase (GSH-Px) in the supernatants were assayed. Reactive oxygen species (ROS) in 10^6^ cfu/mL chlorine-injured bacteria or not were measured and the fold changes of average ROS between them were calculated.

Furthermore, the oxidative stress-response in chlorine-injured *E. coli*, *P. aeruginosa*, *S. aberdeen* and *E. faecalis* was investigated (Fig. 5). It was found that the production of ROS increased in three injured bacteria (*E. coli*, *P. aeruginosa* and *E. faecalis*) up to 2.3-fold and their ROS levels were evident higher than those in the control. Meanwhile, to protect bacteria from ROS destruction, cellular antioxidant systems, including CAT activity, SOD activity, and GSH-Px activity, were activated, correspondingly. The SOD level increased dramatically up to 4.5-fold in the chlorine-injured bacteria (*p* < 0.01). CAT activity was only expressed in *P. aeruginosa* before chlorine exposure, but all four bacteria expressed it after chlorination, and *S. aberdeen* showed the maximal CAT activity of 8.3 U/10^8^ cfu. No GSH-Px activity was expressed in all untreated bacteria, but chlorine-injured *P. aeruginosa*, *S. aberdeen* and *E. faecalis* had improved GSH-Px activity of up to 11.3 U/10^8^ cfu. All these results indicate that a strong oxidative stress-response occurred in the injured bacteria suffering from NaClO exposure.

### Released RP4 from chlorine-killed ARB horizontally transferred to chlorine-injured bacteria by natural transformation more frequently under the same environmental circumstances

**Fig. 6:**
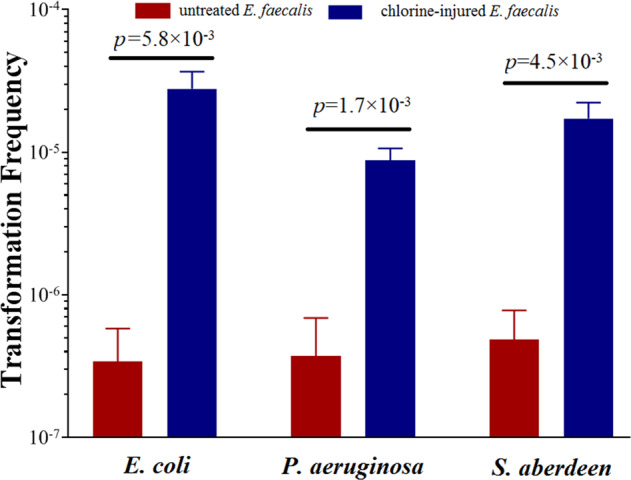
Transformation frequency of RP4 released from killed *Escherichia coli*, *Pseudomonas aeruginosa* and *Salmonella aberdeen* into chlorine-injured *Enterococcus faecalis* or not (10^8^ cfu/mL) naturally at pH 7.2 and 20 °C (*n* = 3; mean ± SD). Suspensions (10^9^ cfu/mL) of *E. coli*, *P. aeruginosa* and *S. aberdeen* (with RP4) treated by 6 mg/L NaClO for 20 min, separately, were filtered to remove ARB and then co-cultured with 10^8^ cfu/mL *E. faecalis* chlorine-injured or not for 60 min (1:10, v/v). Then, transformers on SOB medium containing 50 mg/L Kan, 60 mg/L Amp and 40 mg/L Tet were picked after overnight incubation at 37 °C.

The RP4 released from killed sensitive *E. coli*, *P. aeruginosa* and *S. aberdeen* transformed into chlorine-resistant injury *E. faecalis* naturally, with frequencies of 8.8 × 10^−6^–1.7 × 10^−5^ (Fig. 6). In comparison to the control group, in which *E. faecalis* was untreated by NaClO, the transformation frequencies were improved in the range 37–134-fold (Supplementary Fig. S4).

**Fig. 7:**
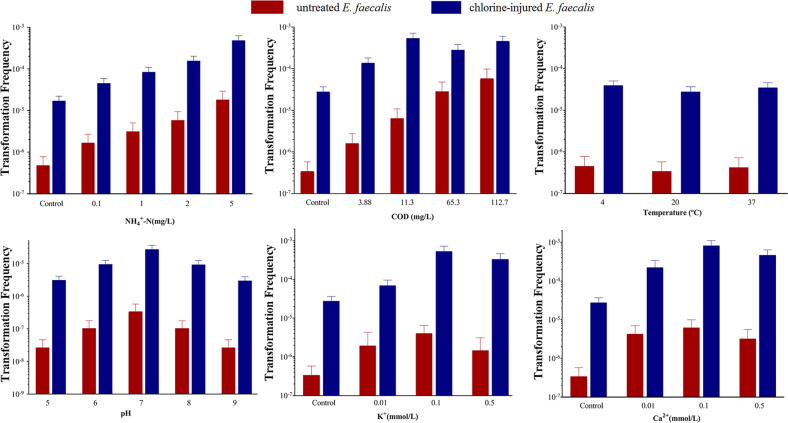
Effect of water quality parameters (NH_4_^+^–N; COD_Mn_; temperature; pH; K^+^; Ca^2+^) on the transfer of RP4 plasmid released from killed *Escherichia coli* to *Enterococcus faecalis* (*n* **=** 3; mean **±** SD). The baseline conditions were as follows: 10^9^ cfu/mL *E. coli* suspension (with RP4) treated by 6 mg/L NaClO for 20 min were filtered to remove ARB and then co-cultured with 10^8^ cfu/mL of chlorine-injured *E. faecalis* under different conditions of water quality parameters for 60 min (1:10, v/v). Then, transformers on SOB medium containing 50 mg/L Kan, 60 mg/L Amp and 40 mg/L Tet were picked after overnight incubation at 37 °C.

To further explore the essential water quality factors on the natural transformation of injured bacteria, the effects of physicochemical parameters, including pH, temperature, COD_Mn_, NH_4_^+^–N and metal ions, on the above-stated transformation frequency were observed (Fig. 7, Supplementary Figs. S5 and S6, Supplementary Text 9). The transformation frequency of released RP4 from killed *E. coli*, *P. aeruginosa* and *S. aberdeen* into injured *E. faecalis* under various physicochemical conditions, revealed that COD_Mn_, NH_4_^+^–N and metal ions (Ca^2+^ and K^+^) could promote the transformation frequency of released RP4 into injured bacteria (*p* < 0.05, *n* = 3, Supplementary Table S6). Above all, transformation frequencies had increased as the concentration of NH_4_^+^–N increased. When it was 5 mg/L, the whole transformation frequency of released plasmid RP4 from killed *E. coli* into injured *E. faecalis* reached around 7.9 × 10^−4^, which was upregulated 55-fold compared with the corresponding injured bacteria without NH_4_^+^–N exposure. Furthermore, the pH value of the water had a great effect on the transformation frequency of released RP4, which reached at most 2.8 × 10^−5^ at pH 7 when RP4 were released from *E. coli*. Then, it would decline with increasing or decreasing pH, reaching zero at pH 10. However, a similar transformation frequency of around 3.0 × 10^−5^ was found when the temperature was in the range 4–37 °C, and there were no significant differences between them (*p* > 0.05, Supplementary Table S6).

## Discussion

Nowadays, antibiotic use, environmental temperature and urban wastewater treatment plants size have been realised as important factors related to resistance persistence and spread in the environment [33, 34]. However, this study is the first to find that chlorine disinfection, which has been used to eliminate pathogens and improve public health in past years [35], increased the frequency of natural transformation and inevitably promoted the horizontal transfer of ARG across bacterial genera via culturable chlorine-injured bacteria. In a sense, chlorine disinfection naturally accelerated the genetic exchange in or across bacterial genera, resulting in the enrichment of ARGs in bacteria after chlorination.

This phenomenon occurred because disinfection of drinking water can help release DNA, including various ARGs and mobile genetic elements (MGEs), such as plasmids, integrons and insertion sequences, from killed donors into the environment [24]. It is more difficult to remove all functional antibiotic-resistant plasmids or ARGs in the ARB than to kill the ARB itself [36, 37]. This study also showed much more NaClO was required to remove transferable plasmids released from killed sensitive ARB. Therefore, using NaClO to decrease bacteria viability is merely fulfilling a necessary demand to “kill” the bacteria. A much higher dose of NaClO should be utilised to destroy plasmids and further break down ARGs in the ARB. Considering that the MGEs involved in the horizontal transfer of ARGs are still quite recalcitrant to disinfection and retain their biological activity [37–39], released ARGs located on MGEs can easily spread via horizontal transfer among species, including human pathogens [40]. Roller et al. reported that even at 6-log inactivation of *Haemophilus influenzae* cells by chlorine dioxide (ClO_2_), the intracellular DNA was largely intact [41]. Here, we further demonstrated that although ARB cells had effectively died following chlorination, their plasmids still retained biological activity and could be available to other bacterial communities via transformation. Hence, when disinfection is performed just to inactivate bacteria or the disinfection dose is not enough, large amounts of functional plasmids or ARGs with transporters are released from the dead bacteria that provide a great opportunity to transfer ARB to other environmental bacteria by natural transformation.

Another important reason explaining the enrichment of ARGs in bacteria after chlorination is that the challenge of disinfection in a factory cannot fully kill all bacteria. It induces survivors with a strong resistance to disinfection to enter a kind of physiologically damaged state in which they can easily capture environmental DNA, including various ARGs and MGEs. A large amount of DNA is released from sensitive bacteria surrounding these injured bacteria, during disinfection, which makes horizontal transfer happen more frequently. In this study, we demonstrated different extents of chlorine resistance among bacterial genera, which then yielded injured bacteria originating from higher resistance populations and, also, it was found that culturable chlorine-injured bacteria can frequently be transformed by antibiotic-resistant plasmids released by killed sensitive ARB. The transformation frequency of these culturable chlorine-injured bacteria increased >550-fold compared with untreated bacteria, resulting in a much higher proportion of iARG in post-disinfection water than that in pre-disinfection water [19]. Generally, competent *E. coli* cells are often used in bacterial transformation owing to their high permeability for bioorganic macromolecules like plasmid DNA [42]. Therefore, the mechanism for increased transformation here can be explained by the fact that the chlorine-injured bacteria also became competent, making plasmid uptake easier, due to the increased cell membrane permeability found in chlorine-injured *E. coli* in this study. Furthermore, the phenomenon of bacterial co-selection of disinfection resistance and antibiotic resistance in tap water found by Khan et al. [16]. might be explained by chlorine-resistant injured bacteria shifting from non-ARB to ARB by natural transformation. In addition, using a chlorine dosage that is lethal to *E. coli* but sub-lethal to *E. faecalis* led to the possibility of producing injured *E. faecalis*, which can uptake antibiotic-resistant plasmids released by dead ARB, resulting in the dissemination of antibiotics resistance across the bacterial genus. Due to *Enterococci* causing biofilm-associated opportunistic infections [30], such as wounds and infective endocarditis, promoting the natural transformation of released plasmids into chlorine-injured *E. faecalis* by the chlorination process may pose a potential risk to public health. Furthermore, the above results indicated that it was not ideal to use *E. coli* to determine the sanitary quality of drinking water, particularly for judging faecal contamination or predicting the possible presence of waterborne pathogens [13, 43], considering that it would result in insufficient bacterial inactivation and the production of culturable chlorine-injured bacteria that show more resistance to chlorination than *E. coli*. These organisms can survive chlorine exposure and enhance ARG transfer across bacterial genera via natural transformation.

Finally, environmental contaminants, COD_Mn_, NH_4_^+^–N and metal ions (Ca^2+^ and K^+^), can enhance the transformation frequency of released plasmid RP4 from killed ARB into culturable chlorine-injured bacteria. In general, the cell competency drastically decreased in the case of planktonic cells when organic nutrients were richly available for cell growth [15]. However, chlorine-injured bacteria still can be induced to become competent in the presence of organic material in the environment, indicating they may also enter into a state of nutritional starvation similar to competent *E. coil* cells existing in the state of biofilm [15]. Ca^2+^ is one of the most typical competence-inducing factors. They are generally considered to induce the formation of pore-like structures on the cell surface for the passing of intact double-stranded DNA [14]. Alkali cations e.g. Na^+^, K^+^ in the presence of PEG6000 were also found all effective for the transformation of *E. coli* [7]. Liu et al. found that the change of total concentration of observed iARGs correlated strongly and positively with the NH_4_^+^–N concentration, during chlorination [19]. Zhang et al. found that a higher concentration of NH_4_^+^–N (15 mg/L) resulted in lower ARGs removal [44]. Therefore, external environmental factors, one of the DNA-uptake mechanisms for bacteria[14], may play important roles in horizontal transfer of ARGs resulting from the chlorination process. It indicated that controlling water quality before disinfection is an effective method to control ARG pollution.

Virtually, the promotion of conjugal transfer between bacteria may also contribute the enrichment of the iARG level in the finished water after chlorination. To determine the effect of chlorine on total transformation events, we also performed an experiment to treat co-cultures of donor (*E. coli* with RP4, Kan-, Amp- and Tet-resistant) and recipient cells (*E. faecalis*, nalidixic acid-resistant) using NaClO (Supplementary Text 10). The results showed (Supplementary Table S7) the number of transformers in viable *E. faecalis* (Kan-, Amp-, Tet- and nalidixic acid resistant) increased 8.3–8.7 folds even after 6–7 mg/L NaClO exposure, comparing with the control group (no chlorine). However, considering no genetic transformation should be occurred under the circumstance of the control (mixture of two kinds of bacteria), conjugal transfer was supposed to occur between *E. coli* and *E. faecalis* in the control. As a result, the increasing number of transformers after the chlorine exposure to mixed bacteria may also come from the promotion of conjugal transfer between bacteria, besides the genetic transformation.

In summary, chlorine-tolerant injured bacteria that are physiologically competent cells present higher plasmid transformation frequency than the corresponding untreated bacteria. Since the transferable plasmids released from killed sensitive ARB have a consistent resistance to degradation through disinfection, the chlorination process can promote the horizontal transfer of the released plasmid into chlorine-injured bacteria through natural transformation and lead to the enrichment of ARGs in viable bacteria. This process was enhanced by environmental contaminants, COD_Mn_, NH_4_^+^–N and metal ions. These results indicate that chlorine disinfection naturally accelerates gene exchange in or between bacterial genera, so that the chlorine-injured opportunistic pathogens can be transferred from non-ARB to ARB through natural transformation during chlorination, which poses a potential risk to disseminate antibiotic resistance in water.

## Supplementary information

Supplementary Information
