# Supplementary material for: Chlorine disinfection promotes the exchange of antibiotic resistance genes across bacterial genera by natural transformation
Source: ISME J. 2020 Apr 23;14(7):1847–56. doi: 10.1038/s41396-020-0656-9 (PMC7305130; doi:10.1038/s41396-020-0656-9)
Supplement: Supplementary file 1 — Supplementary Information [file 41396_2020_656_MOESM1_ESM.docx]

Supplementary Information for:

**Title:** Chlorine disinfection promotes the exchange of antibiotic resistance genes across bacterial genera by natural transformation

**Authors**: Min Jin^1,*^, Lu Liu^1^, Da-ning Wang^1^, Dong Yang^1^, Wei-li Liu^1^, Jing Yin^1^, Zhong-wei Yang^1^, Hua-ran Wang^1^, Zhi-gang Qiu^1^, Zhi-qiang Shen^1^, Dan-yang Shi^1^, Hai-bei Li^1^, Jian-hua Guo^2^, Jun-wen Li^1,*^

^1^Department of Environment and Health, Tianjin Institute of Environmental & Operational Medicine, Key Laboratory of Risk Assessment and Control for Environment & Food Safety, No 1 Dali Road, Tianjin 300050, PR China

^2^Advanced Water Management Centre (AWMC), University of Queensland, St Lucia, Brisbane, QLD 4072, Australia

**^*^Corresponding author:** jinminzh@126.com; junwen9999@hotmail.com

**Supplementary Text 1: Culture conditions of bacteria**

*Escherichia coli* strain ATCC 25922, *Salmonella aberdeen* strain CMCC 50312, *Pseudomonas aeruginosa* strain CMCC 10110 and *Enterococcus faecalis* strain ATCC 33186 were grown separately overnight at 37 ºC in Luria–Bertani broth (BD Diagnostics, USA). Four kinds of selective medium used to culture uninjured bacteria included Endo agar (BD Diagnostics) for *E. coli* 25922, XLD agar (BD Diagnostics) for *Salmonella* 50312, cetrimide agar (BD Diagnostics) for *P*. a*eruginosa* 10110 and CATC agar (BD Diagnostics) for *E. faecalis* 33186. All the viable bacteria in the samples were detected by the TSYA, a kind of repair medium for the injury bacteria. One litre of TSYA contains 15 g agar (Oxoid, UK), 3 g yeast extract (Oxoid) and 30 g TSB (BD Diagnostics). Their corresponding ARB carried by RP4 were isolated and cultured using the above medium with the addition of 50 mg/L kanamycin (Solarbio, China), 60 mg/L ampicillin (Solarbio) and 40 mg/L [tetracycline](http://www.google.com.au/url?sa=t&rct=j&q=&esrc=s&source=web&cd=1&cad=rja&uact=8&ved=0ahUKEwjA2JPdn93MAhVKGJQKHWZtDEwQFgggMAA&url=http%3A%2F%2Fwww.antibioresistance.be%2FTetracycline%2FMenu_Tet.html&usg=AFQjCNFjX2ULabX2dsmx_8-5FVYY5Ww4bg&bvm=bv.122129774,d.dGo) (Solarbio).

**Supplementary Text 2: Preparation of bacterial suspension for exposure to NaClO**

*Escherichia coli* strain ATCC 25922 (RP4), *Salmonella aberdeen* strain CMCC 50312 (RP4), *Pseudomonas* *aeruginosa* strain CMCC 10110 (RP4) and *Enterococcus faecalis* strain ATCC 33186 (RP4) were grown separately overnight at 37 °C in Luria–Bertani broth with the addition of 50 mg/L kanamycin, 60 mg/L ampicillin and 40 mg/L tetracycline. The cells were harvested by centrifugation at 5,000 × *g* for 5 minutes, washed three times with phosphate-buffered saline (PBS), resuspended and adjusted to specific concentrations of 10^5^–10^6^ cfu/mL with PBS. The bacteria were immediately exposed to NaClO.

**Supplementary Text 3: Kinetic modelling of NaClO**

Chlorine decay constants (*k′*) for each experiment were calculated using the Solver function in Microsoft Excel 2007 (Microsoft Corp., USA) to regress the first-order kinetic Eq. (1) using the least-squares method:

*C_t_* = *C_0_*e^-^ *^k′^*^t^ (1)

where *C* and *C_0_* are, respectively, the chlorine residuals (mg/L) at time *t* (min) and time 0.25 minutes (the closest possible measurement to time zero), and *k′* is the first-order disinfectant decay rate constant (per min). *Ct* values were calculated by integrating the residual disinfectant concentration (*C*) up to the given sampling time (*t*).

The natural log values of the survival ratio for each experiment were fit to the efficiency factor Hom (EFH) model using Eq. (2):

lnN_t_/N_0_=-*kC_0_^n^*t^m^[(1-e)/(*nk'*t/*m*)]*^m^* (2)

where *C_0_* and *k′* were determined from Eq. (1), *k* is the inactivation rate constant, *n* is the coefficient of dilution, and *m* is Hom’s exponent. The model’s parameter values were determined by minimising the error sum of squares between the observed and predicted ln(N_t_/N_0_) for disinfection experiments using Microsoft Excel Solver; these were then used to calculate the *Ct* values for the 2-, 3- and 4-log (99.00%, 99.90% and 99.99%) inactivation for NaClO.

**Supplementary Text 4: Concentration and purification of released DNA in disinfected water**

Sodium acetate (3 mol/L, pH 5.2, 1/10 volume) and two volumes of at least 95% ethanol were added to the previously filtered samples collected at the various time-points and then mixed well. After incubation on ice for 1 or 2 hours, all samples were centrifuged at 12,000 × *g* for 30 minutes at room temperature. The supernatant was carefully discarded, and the precipitate was rinsed with 70% ethanol, centrifuged again for 15 minutes and the supernatant was discarded. Afterwards, the pellets were re-dissolved with TE buffers and further purified by a DNA purification kit (Tiangen, Beijing, China).

**Supplementary Text 5: Preparation of chlorine-injured bacteria at a concentration of 10^8^ cfu/mL**

*Escherichia coli*, *Salmonella aberdeen*, *Pseudomonas* *aeruginosa* and *Enterococcus faecalis* were incubated in Luria–Bertani broth overnight at 37 °C, and then various bacterial suspensions with approximate concentrations of 10^9^ cfu/mL were prepared according to Text 2. An experimental NaClO exposure (in the range 4–6 mg/L) was conducted in sterile 250-mL glass bottles containing 100 mL of the above bacteria suspension at 20 °C. After NaClO treatment for 20 minutes, samples were collected to assay both chlorine-injured bacteria and uninjured bacteria. According to the data in Table S2, the final selected treatment doses targeting *E. coli*, *S. aberdeen*, *P*. *aeruginosa* and *E. faecalis* were 4, 5, 4 and 6 mg/L, respectively. The samples were then collected at 20 minutes and adjusted to 10^8^ cfu/mL chlorine-injured bacteria with phosphate-buffered saline.

**Supplementary Text 6: Measurement of *o*-nitrophenyl-β-D-galactopyranoside (ONPG) hydrolysis to assess cell permeability**

*Escherichia coli* grown in 50 mL Luria–Bertani broth for 15 h were transferred to 50 mL of fresh medium containing 7.5 mmol/L isopropyl-β-D-1-thiogalactopyranoside (Sigma–Aldrich, USA) and incubated at 37 °C for 5 hours, with shaking at 150 rpm. Then, the suspension was harvested at 5,000 × *g*, washed five times with phosphate-buffered saline (PBS), and resuspended in PBS solution to prepare the *E. coli* suspension with an approximate concentration of 10^9^ cfu/mL. Under the guidelines in Supplementary Text 5, chlorine-injured *E. coli* at a concentration of 10^8^ cfu/mL was prepared, and then 1 mL of suspension was dispensed into a sterile 1.5-mL tube containing 0.1 mL of 5 mmol/L ONPG (Sigma–Aldrich) as the substrate. The mixture was reacted in a water bath at 37 °C, and then 54 µL of the sample was taken out at 4 minutes intervals for 20 minutes and mixed with 6 μL of 0.2 mol/L Na_2_CO_3_ solution to terminate the reaction between the enzyme and substrate. Absorbance values were determined at 420 nm using a 2550 UV spectrophotometer (Shimadzu, Japan). As controls, 10^7^ and 10^8^ cfu/mL *E. coli* suspensions untreated by NaClO were also observed.

**Supplementary Text 7: Reactive oxygen species (ROS) measurement**

To determine the ROS levels in bacteria, cells were analysed by flow cytometry (BD FACS Calibur, USA) and a DCF-DA/H_2_DCFDA-cellular ROS detection assay kit (Abcam, USA), according to the manufacturer's instructions. Briefly, the bacterial suspensions, chlorine-injured or not, were adjusted to 10^6^ cfu/mL in 0.85% (w/v) saline in a 10-mL tube. Subsequently, 20 μmol/L DCF-DA was added to the suspension and then immediately pre-incubated and protected from light at 37 °C for 45 min. After incubation, the suspension was transferred to a 1.5-mL tube containing antibiotics at room temperature for 1 h. To exclude the background fluorescence intensity of the bacteria, a blank control (with bacteria without any treatment) was established. All samples were detected at an excitation wavelength of 495 nm and an emission wavelength of 520 nm. The ROS levels were analysed using the FlowJo (Tree Star software, San Carlos, CA, USA). As controls, 10^5^ and 10^6^ cfu/mL bacterial suspensions untreated by NaClO were also observed.

**Supplementary Text 8: Detection of bacterial anti-oxidant systems**

Chlorine-injured bacteria at a concentration of 10^8^ cfu/mL were prepared according to Supplementary Text 5, to assay the oxidative stress-response. After sonication at 20 kHz (150 W) for 10 min to break down the bacteria using an ultrasonic cell cracker (VCX750, Sonics, USA), the collected samples were centrifuged at 5,000 × *g*, 4 °C for 3 min. The levels of superoxide dismutase (SOD), catalase (CAT) and glutathione peroxidase (GSH-Px) in the supernatant were all assessed using appropriate kits obtained from the Nanjing Jiancheng Bioengineering Institute (Nanjing, China) by following the manufacturers’ instructions. The absorbance values of each indicator were determined at 405 (CAT), 412 (GSH-PX) and 550 nm (SOD), respectively, using a plate reader (BioTex, USA). As controls, 10^7^ and 10^8^ cfu/mL bacterial suspensions untreated by NaClO were also observed.

**Supplementary Text 9: Measurement of physiochemical parameters in water**

The pH value was determined using a pH meter (PB-10, Sartorius, Germany). The temperature was controlled and measured by a constant temperature magnetic heating agitator (HJ-6B, Jintan, China). Humic acid sodium (0–1 g; Sigma–Aldrich, USA) was spiked into water samples to produce organic contamination, and Chemical Oxygen Demand (COD_Mn_) was then measured using the standard closed reflux–titrimetric method [1]. NH_4_Cl (Sigma–Aldrich) was prepared at 100 mg/L, according to standard methods, and the ammonium nitrogen (NH_4_^+^-N) concentration was measured using a portable colourimeter (DR300, Hach, USA).

**Supplementary Text 10:** **Chlorine treatment of mixture of donors and recipients**

Approximate 10^9^ cfu/mL of *E. coli* (carrier of RP4, donors) and [*E. faecalis*](http://www.baidu.com/link?url=JaXghAaaJEjmQS1vhiob2WF0DJpsdB1E77fX0BCRBa6-pBxvln1ISTP-715c3ssyfUxUQ7UYoebx6sAOnoJdD_&wd=&eqid=e4565ae80006b868000000025d3eed95) (recipient) were prepared according to Supplementary Text 2, respectively. After both of them were mixed (1:1) for 30 minutes in a shaker at 150 rpm (4ºC), they were treated with 5 mg/L, 6 mg/L and 7 mg/L NaClO for 30 min, respectively and then neutralised by 0.1 mol/L of sodium thiosulfate. They were placed at 37 ºC for 1 h. Following this, the mixtures or dilutions were spread on SOB medium containing 50 mg/L Kan, 60 mg/L Amp, 40 mg/L Tet and 6 mg/L nalidixic acid (Nal) to determine the number of [*E. faecalis*](http://www.baidu.com/link?url=JaXghAaaJEjmQS1vhiob2WF0DJpsdB1E77fX0BCRBa6-pBxvln1ISTP-715c3ssyfUxUQ7UYoebx6sAOnoJdD_&wd=&eqid=e4565ae80006b868000000025d3eed95) with RP4 (transformers) or SOB medium with 6 mg/L Nal to determine the number of [*E. faecalis*](http://www.baidu.com/link?url=JaXghAaaJEjmQS1vhiob2WF0DJpsdB1E77fX0BCRBa6-pBxvln1ISTP-715c3ssyfUxUQ7UYoebx6sAOnoJdD_&wd=&eqid=e4565ae80006b868000000025d3eed95). After overnight incubation at 37 ºC, the colonies were counted. Each experiment was performed in triplicate. In parallel, the control without NaClO exposure was also observed to determine the frequency of conjugal transfer. To ensure that the bacteria growing on the SOB medium were transformers([*E. faecalis*](http://www.baidu.com/link?url=JaXghAaaJEjmQS1vhiob2WF0DJpsdB1E77fX0BCRBa6-pBxvln1ISTP-715c3ssyfUxUQ7UYoebx6sAOnoJdD_&wd=&eqid=e4565ae80006b868000000025d3eed95) with RP4) rather than any spontaneous mutations of the recipients or the contamination of donors, at least five colonies were spread on CATC plates of *E. faecalis* containing 50 mg/L Kan, 60 mg/L Amp, 40 mg/L Tet and 6 mg/L Nal, their plasmids were extracted to detect the specific TraG of RP4 by PCR.

1. American Public Health Association (APHA). Standard methods for the examination of water and wastewater. 20th ed. APHA:Washington DC, USA,1998.

**Figure Legends**

**Supplementary Figure S1** Fitted decay curves of NaClO at pH 7.2 and 20 °C during disinfection against ARB with RP4 (*n* = 3; mean ± SD). (**a**) *Escherichia coli*; (**b**) *Pseudomonas* *aeruginosa*; (**c**) *Salmonella aberdeen*; (**d**) *Enterococcus faecalis*.

**Supplementary Figure S2** Fitted inactivation kinetics of ARB (RP4) with NaClO at pH 7.2 and 20 °C when detected by the selective medium. The line represents the best fit of the EFH model to the observed data (*n* = 3; mean ± SD). (**a**) *Escherichia coli*; (**b**) *Pseudomonas aeruginosa*; (**c**) *Salmonella aberdeen*; (**d**) *Enterococcus faecalis.*

**Supplementary Figure S3** Fitted inactivation kinetics of ARB (RP4) with NaClO at pH 7.2 and 20 °C when detected by TSYA. The line represents the best fit of the EFH model to the observed data (*n* = 3; mean ± SD). (**a**) *Escherichia coli*; (**b**) *Pseudomonas aeruginosa*; (**c**) *Salmonella aberdeen*; (**d**) *Enterococcus faecalis.*

**Supplementary Figure S4** Fold change of transformation frequency of RP4 released from killed *Escherichia coli*, *Pseudomonas aeruginosa* and *Salmonella aberdeen* into chlorine-injured *Enterococcus faecalis* or not at pH 7.2 and 20 °C (*n* = 3; mean ± SD).

**Supplementary Figure S5** Effect of water-quality parameters (NH_4_^+^-N; COD_Mn_; temperature; pH; K^+^; Ca^2+^) on the transfer of RP4 plasmid released from killed *Pseudomonas* *aeruginosa* to *Enterococcus faecalis* (*n* = 3; mean ± SD). The baseline conditions were as follows: 10^9^ cfu/mL *Pseudomonas aeruginosa* suspension (with RP4) treated by 6 mg/L NaClO for 20 min were filtered to remove ARB and then co-cultured with 10^8^ cfu/mL of chlorine-injured *E. faecalis* under different conditions of water quality parameters for 60 min (1:10, v/v). Then, transformers on SOB medium containing 50 mg/L Kan, 60 mg/L Amp and 40 mg/L Tet were picked after overnight incubation at 37 °C (
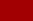
, untreated *E. faecalis*;
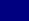
, chlorine-injured *E. faecalis*).

**Supplementary Figure S6** Effect of water-quality parameters (NH_4_^+^-N; COD_Mn_; temperature; pH; K^+^; Ca^2+^) on the transfer of RP4 plasmid released from killed *Salmonella aberdeen* to *Enterococcus faecalis* (*n* = 3; mean ± SD). The baseline conditions were as follows: 10^9^ cfu/mL *Salmonella aberdeen* suspension (with RP4) treated by 6 mg/L NaClO for 20 min were filtered to remove ARB and then co-cultured with 10^8^ cfu/mL of chlorine-injured *E. faecalis* under different conditions of water quality parameters for 60 min (1:10, v/v). Then, transformers on SOB medium containing 50 mg/L Kan, 60 mg/L Amp and 40 mg/L Tet were picked after overnight incubation at 37 °C (
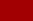
, untreated *E. faecalis*;
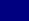
, chlorine-injured *E. faecalis*).

**Table Legends**

**Table S1** Strains and plasmids used in this study.

**Table S2** Average bacteria concentration before and after chlorination with different doses of NaClO for 20 min (*n* = 3; 20 °C, pH 7.2).

**Table S3** Primers used in this study.

**Table S4** Summary of estimated parameters of the fitted EFH model for ARB under exposure to NaClO.

**Table S5** *Ct* value calculated by fitting the EFH model for 2-, 3-, 4- and 5-log inactivation for ARB under exposure to NaClO.

**Table S6** Significant analysis of transformation frequency between chlorine-injured *E. faecalis* under different conditions of water quality parameters and chlorine-injured *E. faecali* in PBS buffer (pH 7.2, 20 °C). *P* values was calculated using Student’s t test.

**Table S7** Effect of NaClO exposure on the occurrence of [*E. faecalis*](http://www.baidu.com/link?url=JaXghAaaJEjmQS1vhiob2WF0DJpsdB1E77fX0BCRBa6-pBxvln1ISTP-715c3ssyfUxUQ7UYoebx6sAOnoJdD_&wd=&eqid=e4565ae80006b868000000025d3eed95) with RP4 in the total [*E. faecalis*](http://www.baidu.com/link?url=JaXghAaaJEjmQS1vhiob2WF0DJpsdB1E77fX0BCRBa6-pBxvln1ISTP-715c3ssyfUxUQ7UYoebx6sAOnoJdD_&wd=&eqid=e4565ae80006b868000000025d3eed95) (n=3).


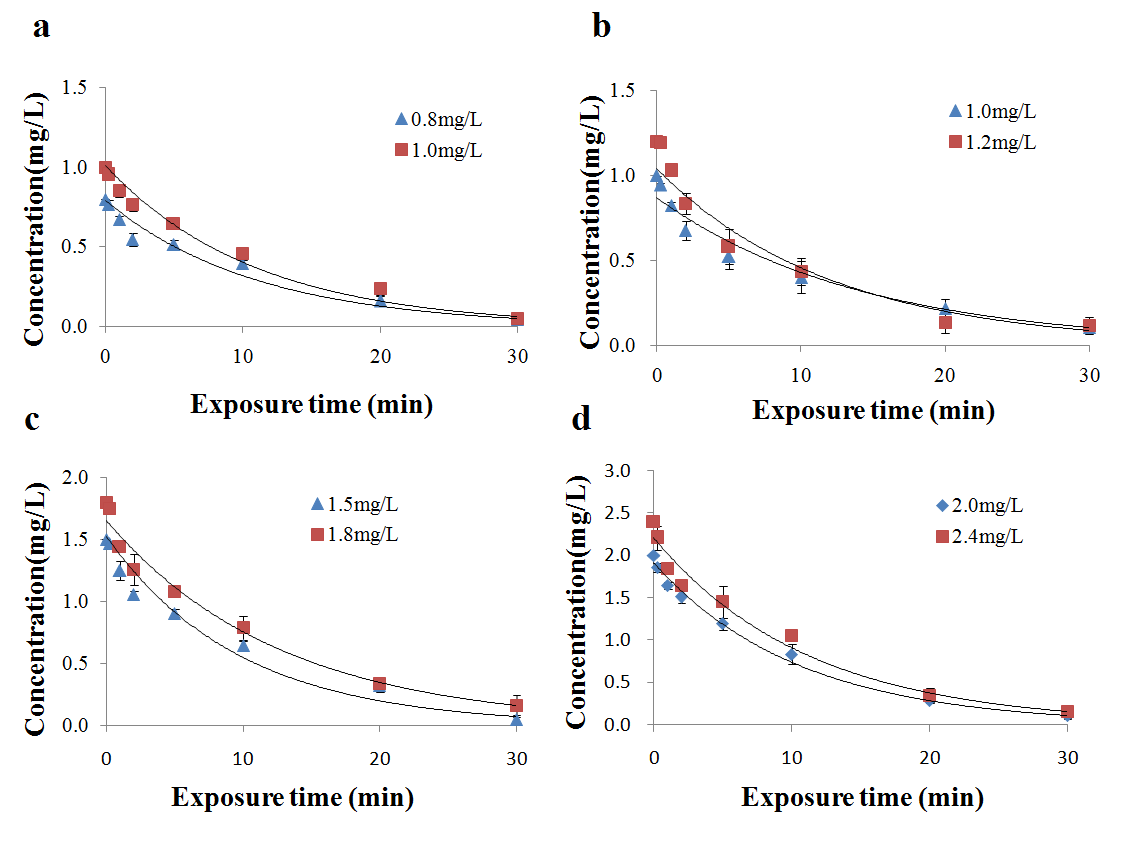


**Supplementary Figure S1**


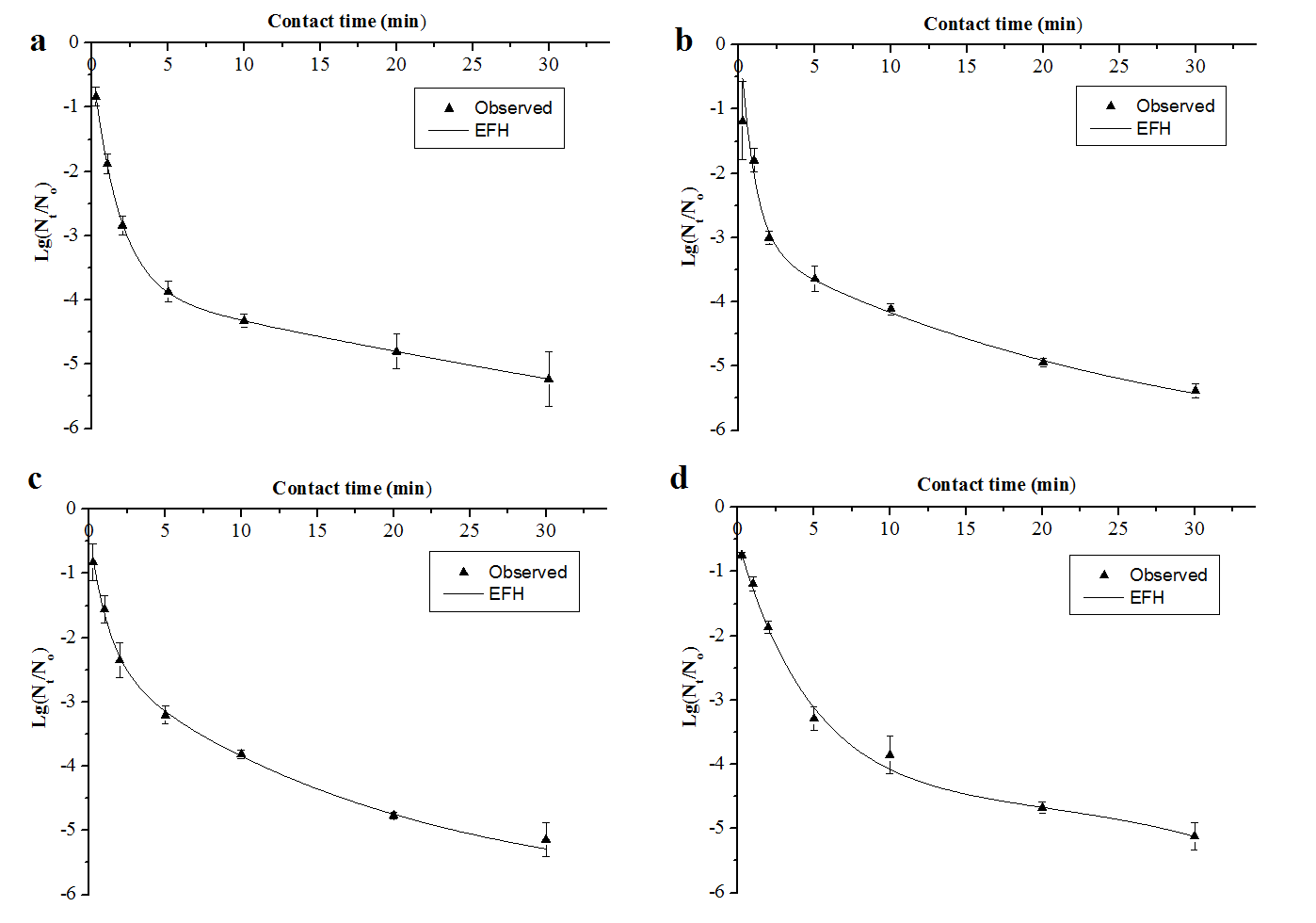


**Supplementary Figure S2**


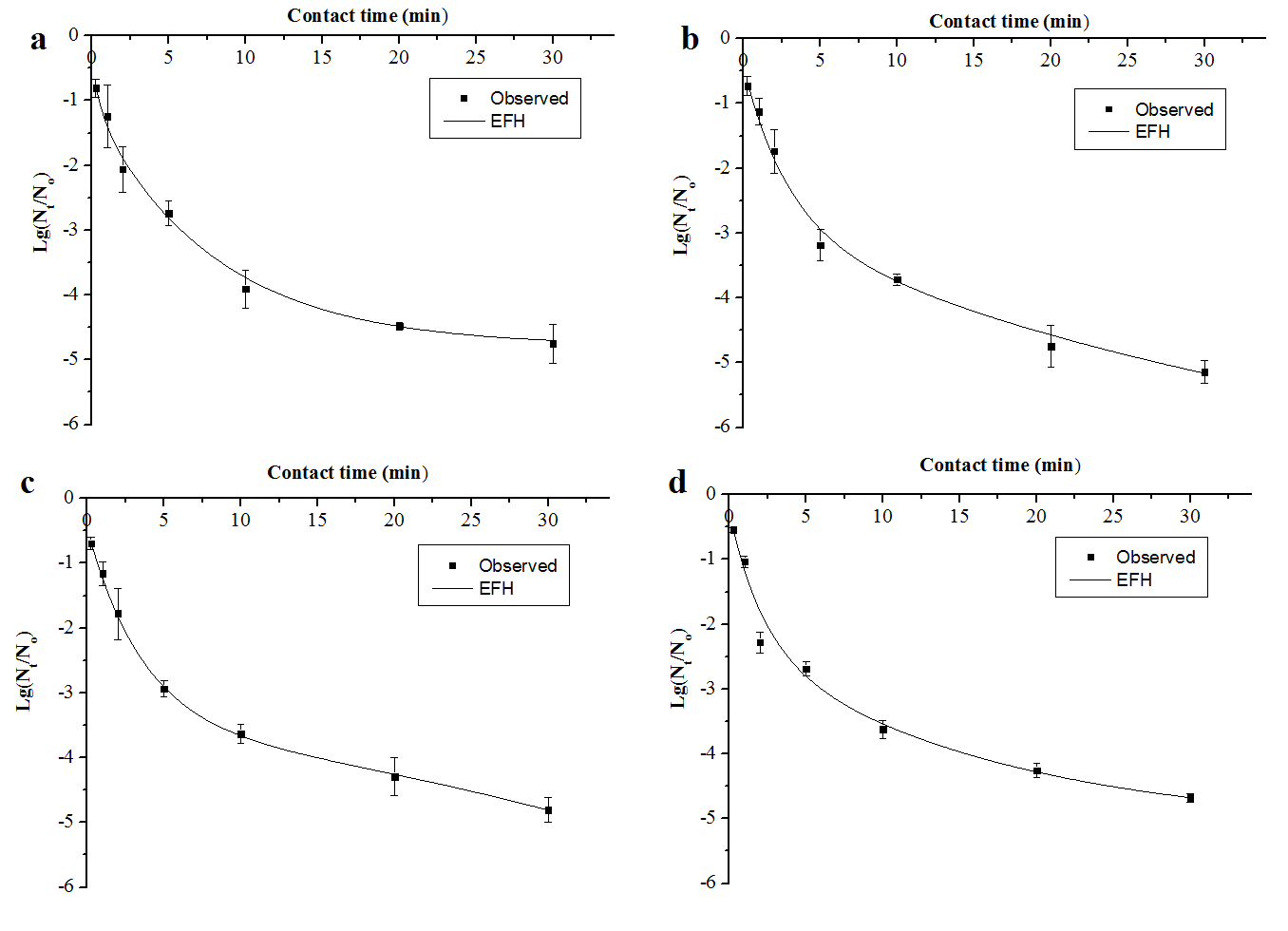


**Supplementary Figure S3**





**Supplementary Figure S4**


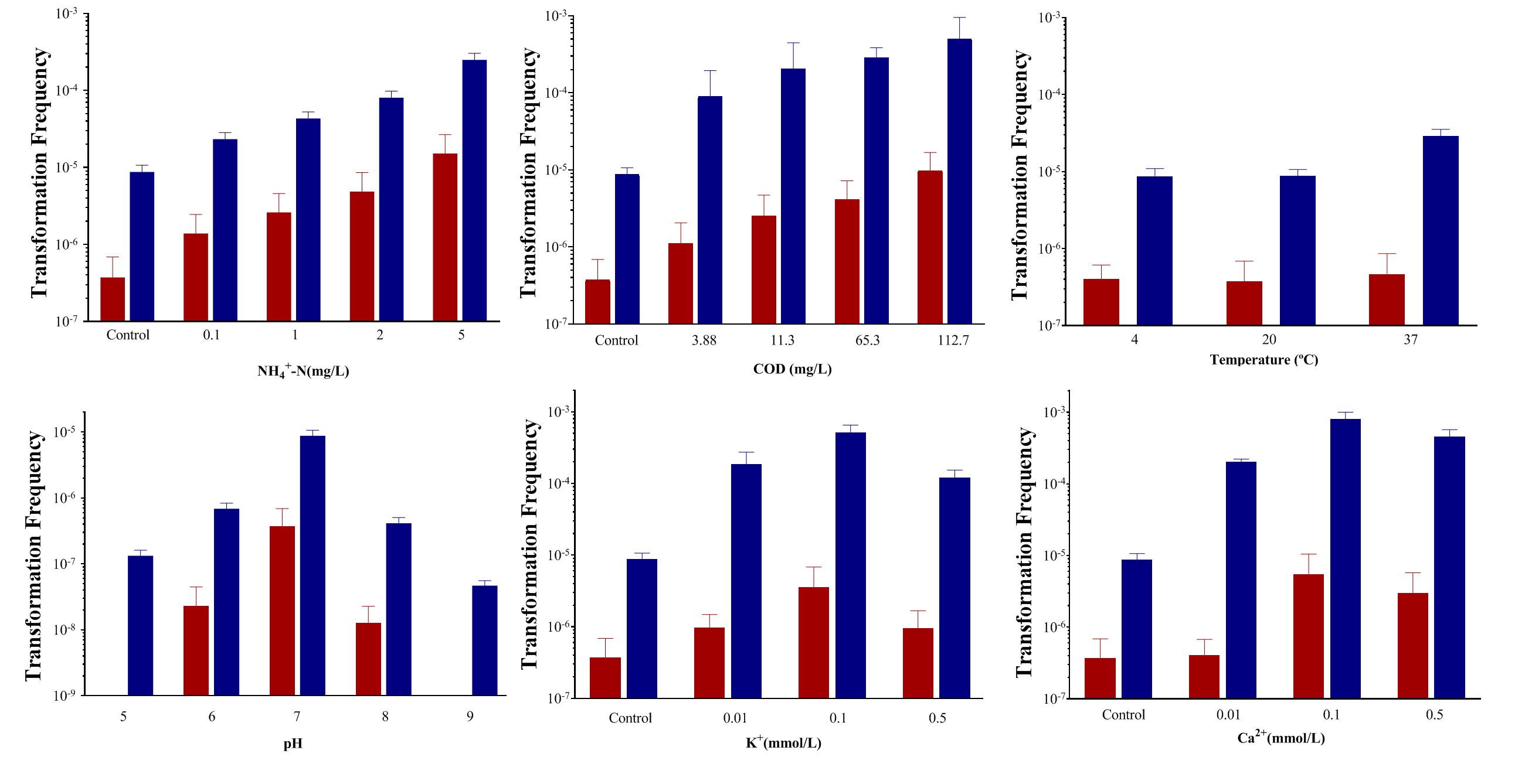


**Supplementary Figure S5**


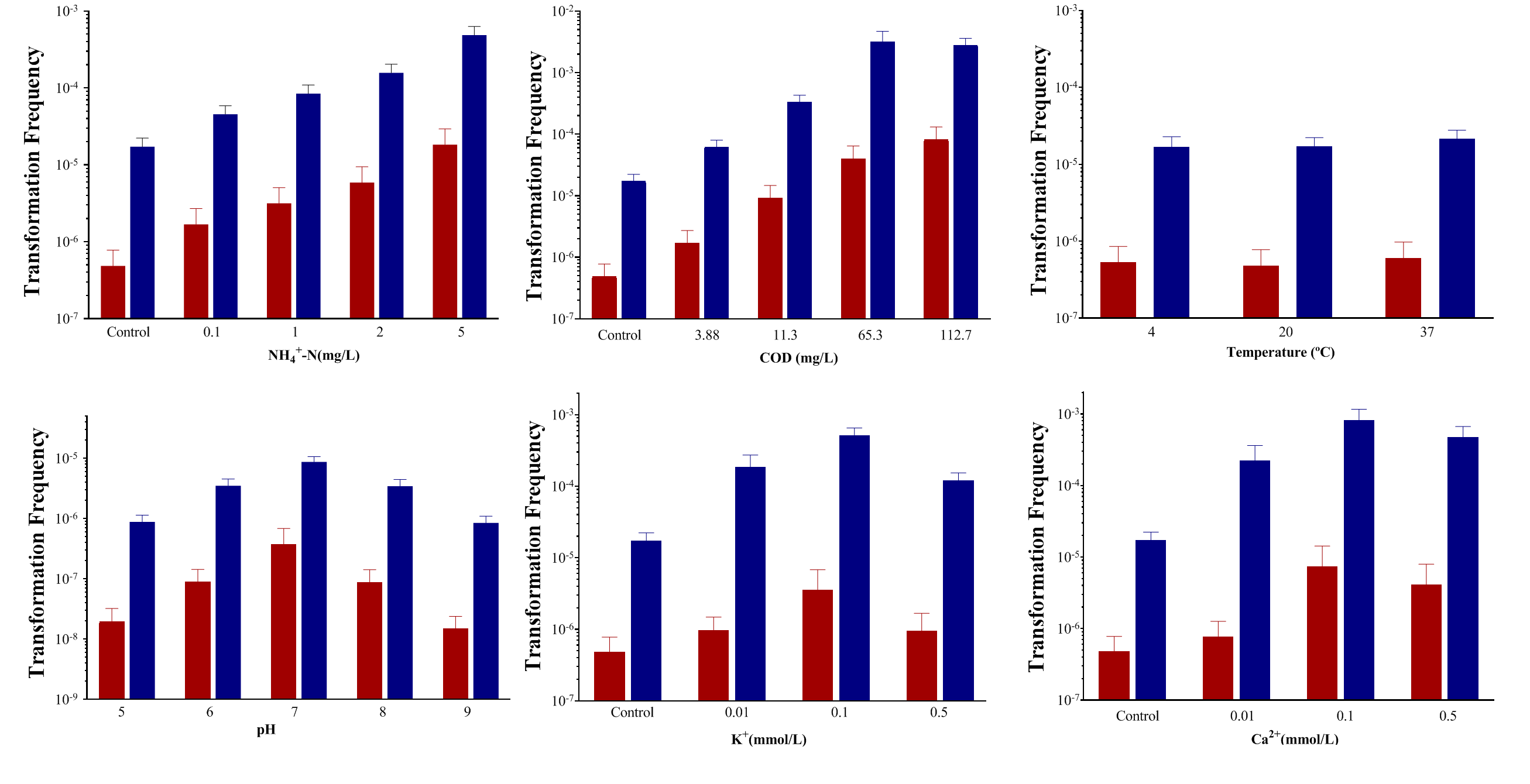


**Supplementary Figure S6**

**Table S1** Strains and plasmids used in this study.

| **Strains and plasmids** | **Description** |
| --- | --- |
| RP4 | Stored in the lab, IncPα plasmid, Kan^r^, Amp^r^, Tet^r^ |
| *E. coli* 25922 | American Type Culture Collection, ATCC |
| *E. faecalis* 33186 | ATCC |
| *S. aberdeen* 50312 | National Center for Medical Culture Collections, China, CMCC, Str^r^ |
| *P*.*aeruginosa* 10110 | CMCC |
| *E. coli* 25922(RP4)* | Stored in the lab, *E. coli* containing RP4 plasmid, Kan^r^, Amp^r^, Tet^r^ |
| *E. faecalis* 33186(RP4)* | Stored in the lab*, E. faecalis* containing RP4 plasmid, Kan^r^, Amp^r^, Tet^r^ |
| *S. aberdeen* 50312(RP4)* | Stored in the lab*, S. aberdeen* containing RP4 plasmid, Kan^r^, Amp^r^, Tet^r^ |
| *P*. *aeruginosa* 10110(RP4)* | Stored in the lab*, P*. *aeruginosa* containing RP4 plasmid, Kan^r^, Amp^r^, Tet^r^ |

**Table S2** Average bacteria concentration before and after chlorination with different doses of NaClO for 20 min (*n* = 3, 20 °C, pH 7.2).

| **Strains** | **Dose**  **(mg/L)** | **Before chlorination**  **(cfu/mL)** | | **After chlorination (cfu/mL)** |  |  |
| --- | --- | --- | --- | --- | --- | --- |
|  |  |  |  | **Injured** |  | **viable*** |
| *E. coli* | 4 | 1.54×10^9^ |  | 1.31×10^8^ |  | 1.63×10^8^ |
|  | 5 | 2.41×10^9^ |  | 1.83×10^6^ |  | 2.25×10^6^ |
|  | 6 | 4.68×10^9^ |  | 1.15×10^3^ |  | 2.95×10^3^ |
|  |  |  |  |  |  |  |
| *P. aeruginosa* | 4 | 2.34×10^9^ |  | 5.02×10^8^ |  | 5.93×10^8^ |
|  | 5 | 3.63×10^9^ |  | 2.53×10^7^ |  | 3.08×10^7^ |
|  | 6 | 6.51×10^9^ |  | 3.52×10^4^ |  | 6.47×10^4^ |
|  |  |  |  |  |  |  |
| *S. aberdeen* | 4 | 2.53×10^9^ |  | 1.46×10^9^ |  | 1.71×10^9^ |
|  | 5 | 1.77×10^9^ |  | 2.77×10^8^ |  | 3.09×10^8^ |
|  | 6 | 3.66×10^9^ |  | 2.88×10^6^ |  | 3.40×10^6^ |
|  |  |  |  |  |  |  |
| *E. faecalis* | 4 | 3.94×10^9^ |  | 2.61×10^9^ |  | 2.97×10^9^ |
|  | 5 | 3.36×10^9^ |  | 1.43×10^9^ |  | 1.72×10^9^ |
|  | 6 | 2.43×10^9^ |  | 6.30×10^8^ |  | 6.51×10^8^ |

*: detected with TSYA media.

**Table S3** Primers used in this study.

| [Target](app:ds:target)s | Sequences  (5ˊ to 3ˊ) | PCR  constitute | PCR  conditions | Product length  (bp) |
| --- | --- | --- | --- | --- |
| TraG | AAAGCGGACAGCATCAGTAACGAA | 2×Master Mix 12.5μl; Primers (10 μM) 1μl; Template 4μl; ddH_2_O 6.5μl. | denaturation:95°C,5min;  30 cycles: 95°C,30s; 55°C,30s;72°C,30s; extension:72°C,10min. | 104 |
|  | GAGCTTGGTGGCCGCATAGTGTAG |  |  |  |

**Table S4** Summary of estimated parameters of the fitted EFH model for ARB under exposure to NaClO.

| Strains | Detection medium | *C_0_^a^* | N_0_^b^ | *k`^c^* | *k^d^* | *n^e^* | m^f^ | | | *R^2^* |
| --- | --- | --- | --- | --- | --- | --- | --- | --- | --- | --- |
| *E. coli* | Endo | 0.8 | 3.6×10^6^ | 0.090 | 5.8032 | 0.9939 | | 0.5191 | 0.9997 | |
|  | TSYA | 1.0 | 3.6×10^6^ | 0.091 | 3.2910 | 0.5657 | | 0.5218 | 0.9963 | |
|  |  |  |  |  |  |  | |  |  | |
| *P. aeruginosa* | CA | 1.0 | 3.8×10^6^ | 0.071 | 4.8116 | 0.3553 | | 0.3661 | 0.9787 | |
|  | TSYA | 1.2 | 3.2×10^6^ | 0.082 | 2.7104 | 0.5658 | | 0.5664 | 0.9886 | |
|  |  |  |  |  |  |  | |  |  | |
| *S. aberdeen* | XDL | 1.5 | 2.8×10^6^ | 0.102 | 3.4421 | 0.2689 | | 0.4319 | 0.9950 | |
|  | TSYA | 1.8 | 3.5×10^6^ | 0.079 | 2.1461 | 0.5783 | | 0.5379 | 0.9986 | |
|  |  |  |  |  |  |  | |  |  | |
| *E. faecalis* | CATA | 2.0 | 3.1×10^6^ | 0.095 | 1.8884 | 1.0313 | | 0.5875 | 0.9967 | |
|  | TSYA | 2.4 | 4.6×10^6^ | 0.089 | 1.8929 | 0.5539 | | 0.5339 | 0.9899 | |

^a^Initial disinfectant concentration, mg/L. ^b^Initial bacterial concentration, cfu/mL. ^c^Average disinfectant decay constant for replicate experiments. ^d^Inactivation rate constant. ^e^Coefficient of dilution. ^f^Hom̀s exponent.

**Table S5** *Ct* value calculated by fitting the EFH model for 2-, 3-, 4- and 5-log inactivation for ARB under exposure to NaClO.

| Strains | Dose (mg/L) | Detection medium | *Ct* value (mg/L·min) | | | | | | | |
| --- | --- | --- | --- | --- | --- | --- | --- | --- | --- | --- |
|  |  |  | 2-lg | | 3-lg | | 4-lg | | 5-lg | |
|  |  |  | Observed | EFH | Observed | EFH | Observed | EFH | Observed | EFH |
| *E. coli* | 0.8 | Endo | >0.76 | 0.82 | >1.46 | 1.65 | >3.26 | 3.68 | >9.31 | 10.88 |
|  |  |  | <1.46 |  | <3.26 |  | <5.61 |  | <12.80 |  |
|  | 1.0 | TSYA | >1.92 | 2.18 | >4.25 | 4.79 | >7.22 | 8.39 | NR^a^ | NR^a^ |
|  |  |  | <4.25 |  | <7.22 |  | <11.79 |  |  |  |
|  |  |  |  |  |  |  |  |  |  |  |
| *P. aeruginosa* | 1.0 | Cetrimide | >0.23 | 0.88 | >1.75 | 1.88 | >4.02 | 6.07 | >12.10 | 12.77 |
|  |  |  | <0.91 |  | <4.02 |  | <7.14 |  | <16.55 |  |
|  | 1.2 | TSYA | >2.09 | 2.36 | >4.73 | 4.93 | >8.30 | 9.86 | >14.03 | 17.74 |
|  |  |  | <4.73 |  | <8.30 |  | <14.03 |  | <19.34 |  |
|  |  |  |  |  |  |  |  |  |  |  |
| *S. aberdeen* | 1.5 | XDL | >1.44 | 2.03 | >2.74 | 5.28 | >10.24 | 11.25 | >16.98 | 19.61 |
|  |  |  | <2.74 |  | <6.03 |  | <16.98 |  | <23.57 |  |
|  | 1.8 | TSYA | >3.21 | 3.74 | >7.28 | 7.74 | >12.75 | 17.26 | NR^a^ | NR^a^ |
|  |  |  | <7.28 |  | <12.75 |  | <21.40 |  |  |  |
|  |  |  |  |  |  |  |  |  |  |  |
| *E. faecalis* | 2.0 | CATC | >3.59 | 3.94 | >7.98 | 7.98 | >7.98 | 13.05 | >22.87 | 29.82 |
|  |  |  | <7.98 |  | <13.71 |  | <13.71 |  | <31.67 |  |
|  | 2.4 | TSYA | >4.24 | 5.05 | >9.53 | 11.05 | >16.52 | 22.81 | NR^a^ | NR^a^ |
|  |  |  | <9.53 |  | <16.52 |  | <27.71 |  |  |  |

^a^Not reached

**Table S6** Significant analysis of transformation frequency between chlorine-injured *E. faecalis* under different conditions of water quality parameters and chlorine-injured *E. faecali* in PBS buffer (pH 7.2, 20 °C). *P* values was calculated using Student’s t test.

| Parameters | Conditions | *E. coli-E. faecalis* | *P. aeruginosa-E. faecalis* | *S. aberdeen-E. faecalis* |
| --- | --- | --- | --- | --- |
| NH_4_^+^-N  (mg/L) | 0.1 | 2.52×10^-2^ | 2.52×10^-2^ | 9.88×10^-3^ |
|  | 1 | 9.53×10^-3^ | 9.53×10^-3^ | 3.25×10^-3^ |
|  | 2 | 6.27×10^-3^ | 6.27×10^-3^ | 2.21×10^-3^ |
|  | 5 | 3.98×10^-3^ | 3.98×10^-3^ | 1.61×10^-3^ |
| COD_Mn_  (mg/L) | 3.88 | 1.31×10^-2^ | 1.40×10^-2^ | 0.251 |
|  | 11.3 | 6.78×10^-3^ | 4.91×10^-3^ | 0.226 |
|  | 65.3 | 9.75×10^-3^ | 2.41×10^-2^ | 8.15×10^-3^ |
|  | 112.7 | 7.13×10^-3^ | 4.24×10^-3^ | 0.127 |
| Temperature  (°C) | 4 | 0.213 | 0.919 | 0.939 |
|  | 37 | 0.443 | 0.391 | 0.696 |
| pH | 5 | 8.79×10^-3^ | 2.11×10^-3^ | 1.45×10^-3^ |
|  | 6 | 2.85×10^-3^ | 1.39×10^-2^ | 1.88×10^-3^ |
|  | 8 | 2.76×10^-2^ | 1.33×10^-2^ | 1.65×10^-3^ |
|  | 9 | 8.64×10^-3^ | 2.07×10^-3^ | 1.41×10^-3^ |
| K^+^  (mmol/L) | 0.01 | 5.91×10^-3^ | 2.78×10^-2^ | 2.38×10^-2^ |
|  | 0.1 | 9.23×10^-3^ | 2.84×10^-3^ | 2.66×10^-3^ |
|  | 0.5 | 1.37×10^-2^ | 1.03×10^-3^ | 4.56×10^-3^ |
| Ca^2+^  (mmol/L) | 0.01 | 4.23×10^-2^ | 6.43×10^-2^ | 5.73×10^-5^ |
|  | 0.1 | 8.56×10^-3^ | 1.54×10^-2^ | 2.69×10^-3^ |
|  | 0.5 | 9.36×10^-3^ | 1.64×10^-2^ | 2.70×10^-3^ |

**Table S7** Effect of NaClO exposure on the occurrence of [*E. faecalis*](http://www.baidu.com/link?url=JaXghAaaJEjmQS1vhiob2WF0DJpsdB1E77fX0BCRBa6-pBxvln1ISTP-715c3ssyfUxUQ7UYoebx6sAOnoJdD_&wd=&eqid=e4565ae80006b868000000025d3eed95) with RP4 in the total [*E. faecalis*](http://www.baidu.com/link?url=JaXghAaaJEjmQS1vhiob2WF0DJpsdB1E77fX0BCRBa6-pBxvln1ISTP-715c3ssyfUxUQ7UYoebx6sAOnoJdD_&wd=&eqid=e4565ae80006b868000000025d3eed95) (n=3).

| Groups | Control  (no chlorine) | NaClO concentration (mg/L) | | |
| --- | --- | --- | --- | --- |
|  |  | 5 | 6 | 7 |
| Total [*E. faecalis*](http://www.baidu.com/link?url=JaXghAaaJEjmQS1vhiob2WF0DJpsdB1E77fX0BCRBa6-pBxvln1ISTP-715c3ssyfUxUQ7UYoebx6sAOnoJdD_&wd=&eqid=e4565ae80006b868000000025d3eed95) (A) | (2.6±0.1) ×10^9^ | (2.6±0.6) ×10^9^ | (2.8±0.5) ×10^9^ | (2.4±0.1) ×10^9^ |
| *E. faecalis* with RP4 (B) | (2.5±0.4) ×10^3^ | (5.3±0.1) ×10^3^ | (2.2±0.9) ×10^4^ | (2.0±0.2) ×10^4^ |
| Ratio(B/A) | 9.6×10^-7^ | 2.0×10^-6^ | 8.0×10^-6^ | 8.3×10^-6^ |
